# Androgen receptor splice variants activating the full-length receptor in mediating resistance to androgen-directed therapy

**DOI:** 10.18632/oncotarget.1802

**Published:** 2014-03-04

**Authors:** Bo Cao, Yanfeng Qi, Guanyi Zhang, Duo Xu, Yang Zhan, Xavier Alvarez, Zhiyong Guo, Xueqi Fu, Stephen R. Plymate, Oliver Sartor, Haitao Zhang, Yan Dong

**Affiliations:** ^1^ Department of Structural and Cellular Biology, Tulane University School of Medicine, Tulane Cancer Center, New Orleans, LA; ^2^ College of Life Sciences, Jilin University, China; ^3^ Department of Pathology and Laboratory Medicine, Tulane University School of Medicine, Tulane Cancer Center, New Orleans, LA; ^4^ Tulane National Primate Research Center, Covington, LA; ^5^ Department of Pharmacology, University of Maryland School of Medicine, Baltimore, MD; ^6^ Departments of Medicine, University of Washington School of Medicine, Seattle, WA; ^7^ Department of Urology, University of Washington School of Medicine, Seattle, WA; ^8^ Department of Urology, Tulane University School of Medicine, Tulane Cancer Center, New Orleans, LA; ^9^ Department of Medicine, Tulane University School of Medicine, Tulane Cancer Center, New Orleans, LA; ^10^ National Engineering Laboratory for AIDS Vaccine, College of Life Sciences, Jilin University, China

**Keywords:** androgen receptor, splice variant, prostate cancer, castration resistance, enzalutamide

## Abstract

Upregulation of constitutively-active androgen receptor splice variants (AR-Vs) has been implicated in AR-driven tumor progression in castration-resistant prostate cancer. To date, functional studies of AR-Vs have been focused mainly on their ability to regulate gene expression independent of the full-length AR (AR-FL). Here, we showed that AR-V7 and AR^v567es^, two major AR-Vs, both facilitated AR-FL nuclear localization in the absence of androgen and mitigated the ability of the antiandrogen enzalutamide to inhibit AR-FL nuclear trafficking. AR-V bound to the promoter of its specific target without AR-FL, but co-occupied the promoter of canonical AR target with AR-FL in a mutually-dependent manner. AR-V expression attenuated both androgen and enzalutamide modulation of AR-FL activity/cell growth, and mitigated the *in vivo* antitumor efficacy of enzalutamide. Furthermore, AR^v567es^ levels were upregulated in xenograft tumors that had acquired enzalutamide resistance. Collectively, this study highlights a dual function of AR-Vs in mediating castration resistance. In addition to trans-activating target genes independent of AR-FL, AR-Vs can serve as a “rheostat” to control the degree of response of AR-FL to androgen-directed therapy via activating AR-FL in an androgen-independent manner. The findings shed new insights into the mechanisms of AR-V-mediated castration resistance and have significant therapeutic implications.

## INTRODUCTION

Androgen deprivation therapy, which disrupts androgen receptor (AR) signaling through androgen ablation or AR antagonists, is the first-line treatment for disseminated prostate cancer. While this regimen is effective initially, progression to the presently incurable and lethal stage, termed castration-resistant prostate cancer (CRPC), invariably occurs [1,2]. Resurgent AR activity is an established driver of therapeutic failure and castration-resistant progression [1,2]. A number of ligand-dependent and –independent mechanisms have been proposed to underlie AR reactivation after androgen-directed therapies [1,2]. For example, overexpression of the full-length AR (AR-FL) was shown to convert prostate cancer growth from a castration-sensitive to a castration-resistant stage [3]. In addition, CRPC tissues were shown to exhibit persistent levels of androgens despite androgen deprivation [1,2]. These led to the development of the potent AR antagonist enzalutamide (MDV3100) and the androgen biosynthesis inhibitor abiraterone for treatment of metastatic CRPC [4,5]. They heralded a new era of prostate cancer therapy. However, many patients presented with therapy-resistant disease, and most initial responders developed acquired resistance within months of therapy initiation, again accompanied by increased prostate-specific antigen (PSA), indicating reactivated AR signaling [4,5]. Emerging evidences indicate that prostate tumors can adapt to these androgen-directed therapies, including the new agents, by signaling through constitutively-active AR splice variants (AR-Vs) that lack the functional ligand-binding domain [6-16].

AR-Vs are upregulated in most CRPCs compared to hormone-naïve cancers [6,7,13-17]. Intriguingly, there is a significant discrepancy between the relative abundance of AR-V mRNAs and that of AR-V proteins in clinical specimens. While the level of AR-V mRNAs is low relative to that of the AR-FL, the AR-V proteins are expressed at a level comparable to that of AR-FL in a considerable portion of metastatic CRPC tissues [6,16]. In addition, the absolute levels of AR-Vs may not be as important as that of AR-FL for their respective activity. This is because AR-FL is located in the cytoplasm in the absence of ligand and translocates to the nucleus and activates target-gene expression upon ligand binding, whereas constitutively-active AR-Vs localize to the nucleus and activate target-gene expression in the absence of ligand [13-15,18-20]. AR-V7 (aka AR3) and AR^v567es^ are two major AR-Vs expressed in clinical specimens [6,7,13-15]. Strikingly, patients with high levels of expression of AR-V7 or detectable expression of AR^v567es^ have a significantly shorter survival than other CRPC patients [6], indicating an association between AR-V expression and a more lethal form of prostate cancer.

Preclinical studies have pointed to an important role of AR-Vs in mediating castration resistance. Ectopic expression of AR-V7 or AR^v567es^ confers castration-resistant growth of LNCaP xenograft tumors [13,15,20]. Conversely, knockdown of AR-V7 attenuates the growth of castration-resistant 22Rv1 xenograft tumors [13]. AR-Vs have also been shown to confer resistance to enzalutamide in preclinical studies. Knockdown of AR-Vs sensitizes 22Rv1 cells and NFκB p52-transfected LNCaP cells to enzalutamide inhibition of growth [8,11]. Reducing AR-V levels with small-molecule drugs improves enzalutamide efficacy against the growth of 22Rv1 cells and xenografts [21]. Thus, AR-V upregulation appears to be a mechanism for prostate cancer cells to evade androgen-directed therapies. A comprehension of mechanisms of AR-V action is paramount for developing effective means to suppress AR-V signaling.

Gene expression profiling showed that AR-Vs regulate the expression of both canonical androgen-responsive genes and a distinct set of targets enriched for cell-cycle function [7,13,15]. The ability of AR-Vs to regulate target-gene expression has been attributed largely to their AR-FL-independent activity [7,8,12-15,19]. However, AR-FL and AR-V7 immunohistochemistry staining of adjacent sections of CRPC specimens showed that AR-V is often co-expressed with AR-FL [7]. We reason that, in addition to binding to chromatin sites and regulating gene expression independent of AR-FL, AR-Vs may bind to chromatin as a complex with AR-FL. Combined, these two activities may account for the expanded AR-V transcriptome. In fact, AR^v567es^ has been shown to coimmunoprecipitate with AR-FL and facilitate AR-FL nuclear localization in the absence of androgen [15]. In the present study, we dissected the interplay between AR-Vs and AR-FL in regulating gene expression and mediating resistance to androgen-directed therapies.

## RESULTS

### AR-V mitigates enzalutamide inhibition of AR-FL nuclear localization

**Figure 1:**
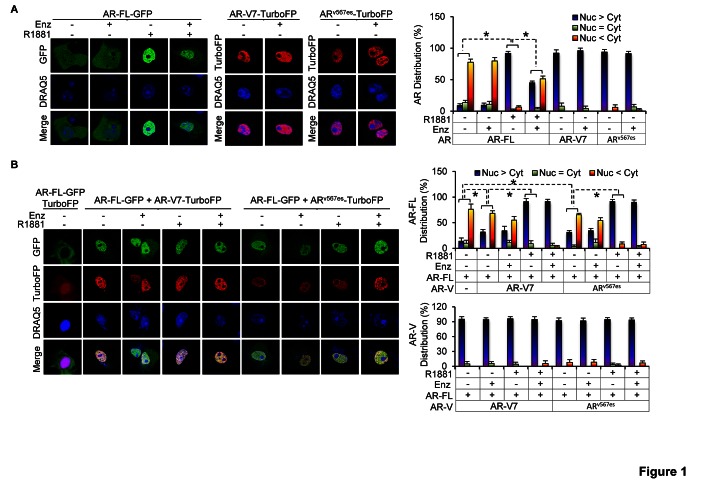
AR-V facilitates AR-FL nuclear localization in the absence of androgen and mitigates enzalutamide inhibition of androgen-induced AR-FL nuclear localization A & B. Confocal fluorescence microscopy of AR-FL and AR-V subcellular localization when expressed alone (A) or when co-expressed with AR-V (B) in COS-7 cells. Right panels, quantitation of % of cells with predominantly nuclear, equally nuclear and cytoplasmic, or predominantly cytoplasmic expression. DRAQ5, nuclear stain. Cells cultured in androgen-deprived condition were pre-treated with 10 μM enzalutamide (Enz) for 2 hr, followed by treatment with or without 1 nM R1881 for 3 hr. *, *P* < 0.05.

Both AR^v567es^ and AR-V7 can reside constitutively in the nucleus [14,15,18], and AR^v567es^ has been shown to facilitate AR-FL nuclear localization in the absence of androgen [15]. Enzalutamide is known to attenuate androgen-induced AR-FL nuclear localization in cells expressing AR-FL alone [22]. To assess the effect of AR-V7 on AR-FL subcellular localization and the impact of AR-Vs on enzalutamide modulation of AR-FL localization, we expressed AR-FL-green-fluorescent-protein (AR-FL-GFP) with or without AR-V7-turbo-red-fluorescent-protein (AR-V7-TurboFP) or AR^v567es^-TurboFP in the AR-null COS-7 cells. Consistent with previous reports [14,15,18], as shown in Figure 1A, both AR-Vs were found primarily in the nucleus, whereas AR-FL localized predominantly in the cytoplasm in androgen-deprived conditions. Enzalutamide caused ~50% reduction of androgen-induced AR-FL nuclear localization, but had no effect on AR-V localization or AR-FL localization in the absence of androgen.

When co-expressed with AR-V7 or AR^v567es^ (Figure 1B), AR-FL could localize to the nucleus in the absence of androgen. The nuclear localization was unaffected by enzalutamide. Strikingly, although addition of androgen further induced AR-FL nuclear localization, enzalutamide could not retain AR-FL in the cytoplasm when AR-V was present. Moreover, AR-V localization was not affected by androgen or enzalutamide even when co-expressed with AR-FL. A similar result was obtained in the PC-3 prostate cancer cells (Supplementary Figure 1). Taken together, the data suggest that AR-Vs facilitate AR-FL nuclear localization in the absence of androgen and mitigate the ability of enzalutamide to inhibit androgen-induced AR-FL nuclear localization.

### AR-V and AR-FL co-occupy the target-gene promoter

**Figure 2:**
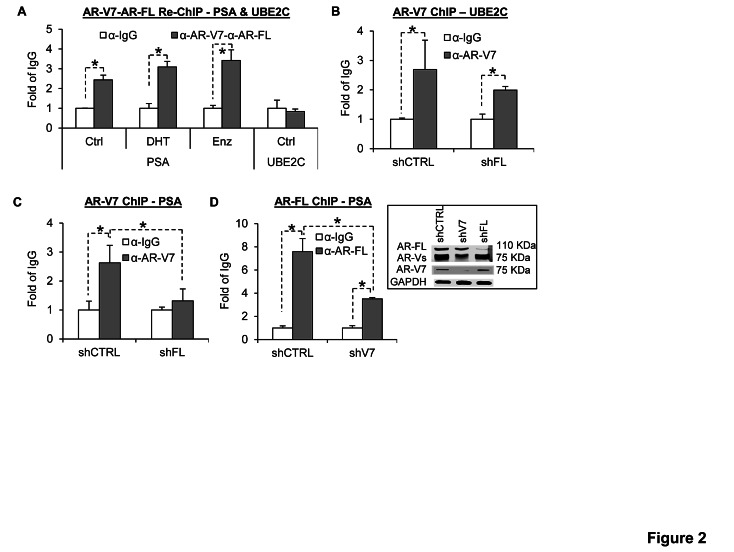
AR-V7 and AR-FL co-occupy the PSA, but not UBE2C, promoter in a mutually dependent manner A. Sequential ChIP analysis in 22Rv1 cells with an AR-V7 antibody followed by an AR-FL antibody showing co-occupancy of the PSA, but not UBE2C, promoter by AR-V7 and AR-FL. Enzalutamide (Enz), 10 μM. DHT, 1 nM. B. AR-V7 ChIP analysis in 22Rv1 cells showing AR-V7 binding to the UBE2C promoter. C. AR-V7 ChIP analysis in 22Rv1 cells showing AR-FL knockdown diminishes AR-V7 binding to the PSA promoter. D. AR-FL ChIP analysis in 22Rv1 cells showing AR-V7 knockdown reduces AR-FL binding to the PSA promoter. The values of the IgG samples are set as 1, and the ChIP results are presented as relative fold of IgG. *, *P* < 0.05. Western blots showed the knockdown efficacy of AR-FL and AR-V7.

Although AR-V-mediated AR-FL nuclear localization may not necessarily entail a physical interaction between AR-V and AR-FL, AR^v567es^ has been shown to coimmunoprecipitate with AR-FL, indicating AR-V can form a complex with AR-FL [15]. To find out whether they bind to target promoters as a complex, we performed sequential chromatin immunoprecipitation (Re-ChIP) analysis with an AR-V7 antibody followed by an AR-FL antibody in 22Rv1 cells, which express endogenous AR-V7 and are in part driven by AR-V7 [23]. We had to limit the analysis to AR-V7 because it is the only AR-V to which a specific antibody has been developed. As shown in Figure 2A, we detected co-occupancy of AR-V7 and AR-FL on the promoter of the PSA gene, and the co-occupancy was unaffected by androgen or enzalutamide treatment. In contrast, the promoter of ubiquitin-conjugating enzyme E2C (UBE2C) is only bound by AR-V7 (Figure 2A and 2B), and ChIP assay showed that AR-FL knockdown (shFL) did not significantly affect the binding (Figure 2B). This is consistent with UBE2C as an AR-V-specific target [6,7]. We then conducted a ChIP assay on the PSA promoter in 22Rv1 cells with or without specific knockdown of AR-FL or AR-V7 in androgen-deprived condition. As shown in Figure 2C, AR-FL knockdown diminished AR-V7 binding to the PSA promoter. Similarly, AR-V7 knockdown (shV7) reduced androgen-independent AR-FL binding to the promoter (Figure 2D). Collectively, the data indicate that, in the absence of androgen, AR-V and AR-FL co-occupy the promoter of canonical androgen-responsive gene, but not AR-V-specific target, in a mutually-dependent manner.

### AR-V attenuates androgen-induced AR-FL transactivation

**Figure 3:**
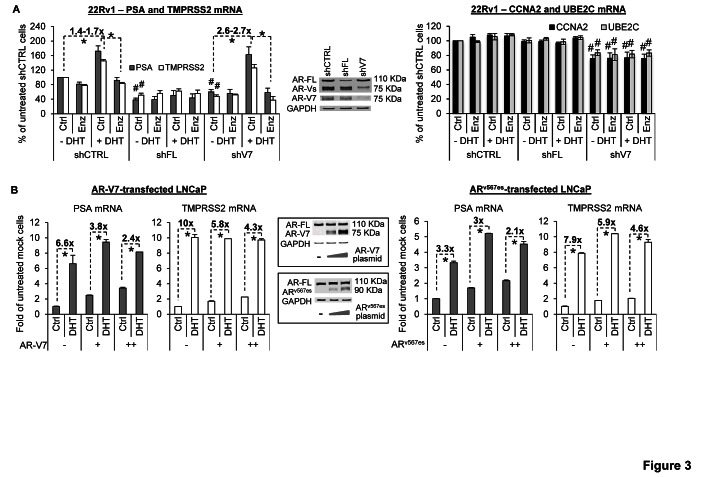
AR-V attenuates androgen and enzalutamide modulation of AR-target expression A. qRT-PCR analysis showing reduced androgen-independent expression of PSA and TMPRSS2 after knockdown of either AR-FL or AR-V7 (left panel) and reduced expression of CCNA2 and UBE2C only after AR-V7 knockdown (right panel). AR-V7 knockdown also renders 22Rv1 cells more sensitive to DHT and enzalutamide modulation of PSA and TMPRSS2 expression. B. qRT-PCR analysis showing that AR-V transfection dose-dependently attenuates DHT induction of PSA and TMPRSS2 in LNCaP cells. Treatment duration, 8 hr (A); 4 hr (B). Enzalutamide (Enz), 10 μM. DHT, 1 nM. *, *P* < 0.05. #, *P* < 0.05 from untreated control-shRNA cells.

To determine the impact of promoter co-occupancy on target gene expression, we measured the mRNA levels of both canonical androgen-responsive genes (PSA and TMPRSS2) and AR-V-specific targets (CCNA2 and UBE2C) in 22Rv1 cells in response to AR-FL or AR-V7 knockdown (Figure 3A). While knockdown of AR-FL and AR-V7 both reduced androgen-independent expression of PSA and TMPRSS2, only AR-V7 knockdown downregulated CCNA2 and UBE2C. Notably, although AR-V7 knockdown diminished basal PSA and TMPRSS2 levels, the levels after androgen stimulation were essentially the same in control and AR-V7-knockdown cells. AR-V7 knockdown thus led to a higher magnitude of androgen induction of PSA (2.7-fold *vs*. 1.7-fold) and TMPRSS2 (2.6-fold *vs*. 1.4-fold), and enzalutamide was very effective in blocking the induction. Conversely, ectopic expression of AR-V7 or AR^v567es^ in LNCaP cells dose-dependently induced basal PSA and TMPRSS2 expression and diminished the degree of response of PSA and TMPRSS2 to androgen (Figure 3B and Supplementary Figure 2). Taken together, the data indicate that, in addition to *trans*-activating a distinct set of genes, AR-Vs activate AR-FL in an androgen-independent manner to induce the expression of their shared targets. In doing so, AR-Vs could serve as “rheostats” to control the degree of response of AR-FL to androgen and to androgen-directed therapy. Interestingly, while ectopic co-expression of AR-V7 or AR^v567es^ rendered enzalutamide ineffective against androgen-induced AR-FL nuclear localization (Figure 1B), the presence of AR-V7 did not affect the ability of enzalutamide to inhibit androgen-dependent expression of PSA and TMPRSS2 (Figure 3A and Supplementary Figure 2). Collectively, these results suggest that AR-Vs could facilitate the nuclear localization of AR-FL in the presence of enzalutamide, but are unable to overcome the suppression of ligand-activated AR-FL transactivation by enzalutamide.

### AR-V mitigates androgen and enzalutamide modulation of cell growth

**Figure 4:**
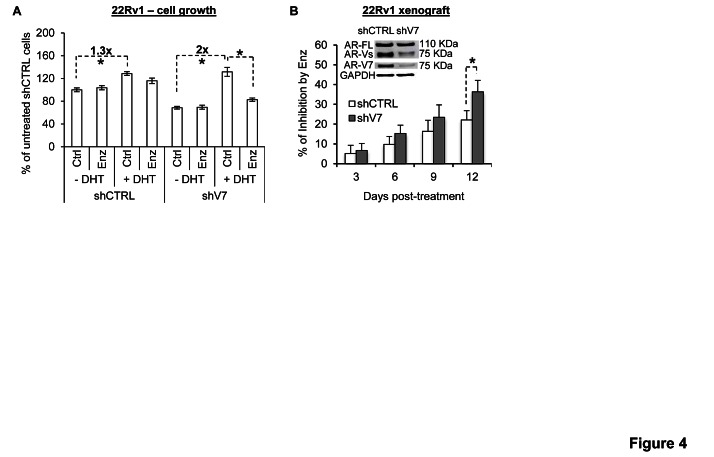
AR-V attenuates androgen and enzalutamide modulation of cell growth A. AR-V7 knockdown enhances the response of 22Rv1 cells to androgen and enzalutamide modulation of cell growth. B. Enzalutamide inhibition of 22Rv1 tumor growth becomes more pronounced after AR-V7 knockdown. Data are expressed as % of inhibition by enzalutamide. *, *P* < 0.05. Enzalutamide (Enz), 10 mg/kg/day. n = 8.

We proceeded to characterize the effect of AR-V7 knockdown on androgen and enzalutamide modulation of the growth of 22Rv1 cells. Congruent with the mRNA data, after AR-V7 knockdown, the cells became more sensitive to DHT induction of growth (Figure 4A; ~2-fold in AR-V7-knockdown cells *vs.* 1.3-fold in control cells). Consequently, the knockdown cells were more responsive to enzalutamide growth inhibition than the control cells. We next inoculated AR-V7-knockdown cells or control cells in nude mice, and characterized the response of the ensuing tumors to enzalutamide. As shown in Figure 4B, growth inhibition by enzalutamide was more pronounced after AR-V7 knockdown (the tumor growth curves are presented in Supplementary Figure 3). Collectively, the data suggest that AR-V may contribute to enzalutamide resistance by dampening the response of the cells to androgen induction of growth.

### Increased AR-Vs in tumors that had developed acquired resistance to enzalutamide

**Figure 5:**
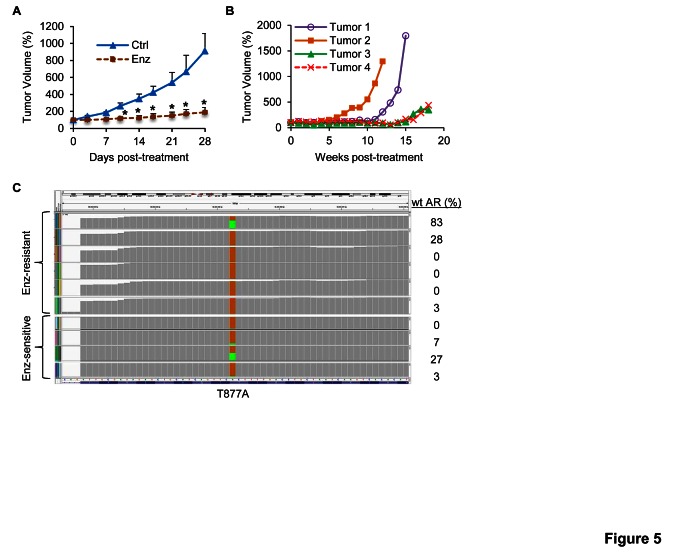
Absence of AR F876L mutation in LNCaP tumors that have developed acquired resistance to enzalutamide A. Enzalutamide (Enz) inhibits the growth of castration-resistant LNCaP tumors initially. LNCaP cells were transduced with lentivirus encoding wild-type (wt) AR-FL before inoculated into castrated mice. *, *P* < 0.05 from the control group. n = 5. B. LNCaP tumors resume growth after 7-17 weeks of enzalutamide treatment. The mean tumor volumes were presented as % of original tumor size at Day 0 of treatment. C. Integrative Genomics Viewer (IGV) plot of RNA-seq data showing no detection of F876L mutation in the AR gene in enzalutamide-sensitive and –resistant LNCaP tumors. The brown boxes represent the relative frequencies of T877A-mutated AR that is present in the LNCaP tumors. The relative frequencies of the transduced wt AR remained in the tumors are denoted by the green boxes and tabled on the right. Allele frequency threshold was set at 0.01.

**Figure 6:**
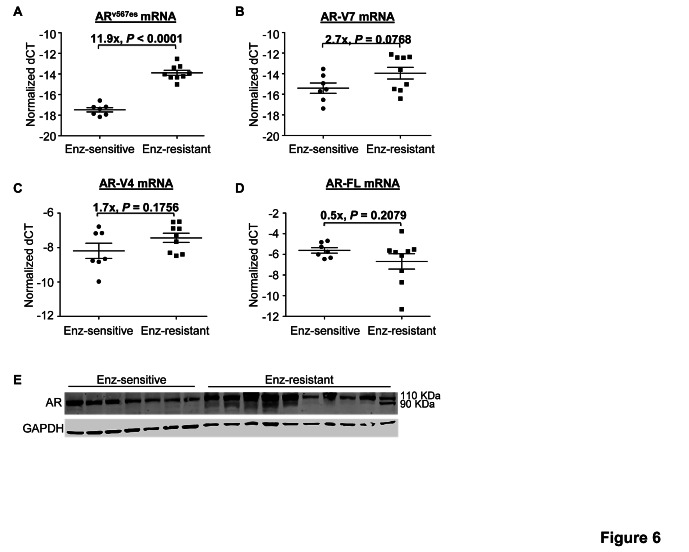
Increased AR-V expression in LNCaP tumors that have developed acquired resistance to enzalutamide A-D. qRT-PCR analysis of the levels of AR-V transcripts. Fold changes are calculated from the difference in mean ∆C_T_ between the enzalutamide-sensitive and enzalutamide-resistant groups (2^∆∆CT^). E. Western blot analysis of the levels of AR-FL and AR-V proteins.

Enzalutamide has been demonstrated to be very effective against the growth of castration-resistant AR-FL-overexpressing LNCaP xenografts [22]. As shown in Figure 5A, we observed the same phenomenon in xenografts established by inoculating LNCaP cells that were transduced with wild-type-AR-FL-encoding lentivirus into castrated nude mice. Some tumors resumed growth with prolonged treatment (after 7-17 weeks) (Figure 5B). We serially passaged the relapsed Tumor #1 and #2 (Figure 5B) in castrated mice treated with enzalutamide, and considered tumors from the second to fourth passages as enzalutamide resistant. RNA-seq analysis of four enzalutamide-sensitive tumors and six enzalutamide-resistant tumors showed that none of the tumors carried the AR F876L missense mutation (Figure 5C), which was identified in enzalutamide-resistant LNCaP cells and shown to confer agonist activity to enzalutamide [24-26]. Instead, the transcripts of AR^v567es^ and AR-V7 (trending toward significance) were upregulated in enzalutamide-resistant tumors, while the levels of AR-V4 or AR-FL transcript did not differ (Figure 6A-D). The upregulation of AR-V was also reflected at the protein level (Figure 6E). Interestingly, all the enzalutamide-resistant tumors that showed higher AR-V protein expression also express increased levels of glucocorticoid receptor (Supplementary Figure 4), the upregulation of which has been shown to be a mechanism of acquired resistance to enzalutamide [27]. The data indicate that these tumors may use multiple mechanisms to evade enzalutamide treatment.

## DISCUSSION

To date, the ability of AR-Vs to contribute to castration resistance has been attributed largely to their AR-FL-independent constitutive activity in regulating gene expression. Here, we identified what we believe to be a novel mechanism of AR-V action. We showed that AR-V7 and AR^v567es^, two major AR-Vs, not only facilitate AR-FL nuclear localization in the absence of androgen but also mitigate the ability of the antiandrogen enzalutamide to inhibit androgen-induced AR-FL nuclear localization. In the nucleus, AR-V7 binds to the promoter of its specific target without AR-FL, but co-occupies the promoter of canonical androgen-responsive gene with AR-FL in a mutually-dependent manner. The co-occupancy is not affected by androgen or enzalutamide. Concordantly, knockdown of AR-FL and AR-V7 both result in reduced androgen-independent expression of canonical androgen-responsive genes, but only AR-V7 knockdown downregulates AR-V-specific targets. Notably, although basal levels of canonical androgen-responsive genes are diminished after AR-V7 knockdown, or elevated after AR-V7 or AR^v567es^ overexpression, the levels after androgen stimulation are unaffected. Thus, AR-Vs appear to repress the degree of response of AR-FL to androgen by activating AR-FL to induce target expression in an androgen-independent manner. This is further supported by the improved sensitivity of the cells to androgen induction of cell growth and enzalutamide inhibition of cell growth after AR-V7 knockdown. These collective findings suggest that, in addition to AR-FL-independent constitutive transactivation, AR-Vs may serve as “rheostats” to control the degree of response of AR-FL to androgen and to androgen-directed therapy.

In the present study, we also showed that enzalutamide becomes more potent in thwarting the growth of 22Rv1 xenograft tumors after AR-V7 knockdown, indicating that targeting both AR-Vs and AR-FL is needed to achieve complete AR blockade. While corroborating the *in vitro* observations from Li *et al*. [8] and Nadiminty *et al.* [11], the data contrast the finding from Watson *et al.* that ectopic expression of AR-V7 in AR-FL-overexpressing LNCaP xenograft tumors does not affect the growth inhibitory efficacy of enzalutamide [20]. A plausible explanation for the discrepancy is that, in the context of AR overexpression, the growth of LNCaP tumors may be driven mainly by the AR-FL signaling, making enzalutamide highly effective irrespective of AR-V expression. Nonetheless, we showed that, when the ectopically-expressed AR-FL is lost in these tumors, they can become resistant to enzalutamide. The resistance is accompanied by increased expression of AR^v567es^. Thus, these tumors may also evade enzalutamide treatment through shifting towards AR-V-mediated signaling.

The significance of our finding that AR-Vs activate AR-FL to induce target-gene expression in an androgen-independent manner is based on the premise that AR-Vs and AR-FL are often co-expressed in biological contexts. This is supported by overlapping AR-FL and AR-V7 immunohistochemistry staining of adjacent sections of CRPC specimens [7]. This is also supported by the finding that androgen deprivation coordinately increases AR-FL and AR-V mRNAs by inducing the transcription of the AR gene and thereby increasing the recruitment of splicing factors to AR pre-mRNA to splice both AR-FL and AR-V mRNAs [9]. AR-V expression may also be a result of AR gene rearrangements [28,29], and gene-arrangement-caused AR-V production appears to occur at the expense of AR-FL [29]. However, a clonal selection process is required for gene-rearrangement-mediated AR-V production to be manifested at the level of tumor tissues. This appears to be in contrast to the rather rapid change of AR-V levels observed in xenograft tumors after androgen ablation or androgen replacement [15,20]. Further, different AR-Vs can be expressed in the same tissues. Clonal expansion of cells with one type of gene arrangement could lead to expression of one specific AR-V but may not be able to account for the expression of different AR-Vs. Finally, our data showing co-occupancy of AR-V7 and AR-FL on the PSA promoter in a mutually-dependent manner and increased response of AR-FL to androgen after AR-V7 knockdown provided further support to the co-expression of AR-FL and AR-V in the same cells. Thus, the ability of AR-Vs to activate AR-FL in an androgen-independent manner could be as important as their AR-FL-independent *trans*-activating activity in mediating castration resistance.

Our finding of AR-V and AR-FL co-regulating the expression of canonical androgen-responsive genes in androgen-deprived condition is reminiscent of the transcriptome data from Hu *et al*. that knockout of AR-FL in AR-V-transfected LNCaP cells almost completely abolishes the expression of at least a subset of canonical androgen-responsive genes [7]. In addition to regulating canonical androgen-responsive genes, AR-Vs have also been shown to regulate a distinct set of targets enriched for cell-cycle function [6,7,13]. This is further corroborated by our ChIP data showing the promoter of UBE2C is bound by AR-V7 but not AR-FL. Receptor dimerization is a crucial step of AR-FL activation [30]. AR^v567es^ has been shown to co-immunoprecipitate with AR-FL [15]. Here, we showed that AR-V7 and AR-FL co-reside on the promoter of their shared target. AR-V7 and AR^v567es^ can localize constitutively to the nucleus, and facilitate AR-FL nuclear localization in the absence of androgen. It is therefore possible that AR-V7 and AR^v567es^ dimerize with AR-FL in the cytoplasm in an androgen-independent manner, and the heterodimer translocates to the nucleus and binds to regulatory elements of their shared targets to regulate the transcription of these targets. It remains unknown as to whether dimerization is required for AR-Vs to regulate their specific targets. Future studies are needed to define the dimeric nature of AR-Vs in regulating gene expression.

In summary, our study provides further evidence to support AR-V upregulation as a means for prostate cancer cells to evade all androgen-directed therapies currently accepted in the clinic. Mechanistically, we identified a novel mechanism by which AR-Vs mediate castration-resistant progression. We showed that AR-Vs can activate AR-FL to induce target expression in an androgen-independent manner. By doing so, AR-Vs may serve as “rheostats” to control the degree of response of AR-FL to androgen and to androgen-directed therapy. Since AR-Vs are often co-expressed with AR-FL in biological contexts, this mechanism of AR-V action may be equally important as its AR-FL-independent activity to castration resistance. These findings underscore a critical need to develop effective means to target both AR-Vs and AR-FL to achieve complete AR blockade for more effective combat of these clinically challenging tumors. Several natural or synthetic compounds have been shown pre-clinically to inhibit AR-V and AR-FL actions [17,21,31-35]. Proof of efficacy in clinical trials is keenly awaited.

## METHODS

### Cell Lines and Reagents

LNCaP, 22Rv1, COS-7, and PC-3 cells were obtained from American Type Culture Collection at Passage 4. Cells used in this study were within 20 passages (~3 months of non-continuous culturing). All cell lines were tested and authenticated by the method of short tandem repeat profiling. Enzalutamide was purchased from Selleck Chemicals (Houston, TX), and the purity of >99% was confirmed by Nuclear Magnetic Resonance. The following antibodies were used in Western blot analysis: anti-glyceraldehyde-3-phosphate dehydrogenase (GAPDH, Millipore), anti-AR (N-terminus-directed; PG-21, Millipore), and anti-AR-V7 (Precision Antibody). Cell growth was determined by the Sulforhodamine assay.

### Subcellular Localization

AR subcellular localization is detected by confocal fluorescence microscopy. The pTurboFP-AR-V7 and pTurboFP-AR^v567es^ plasmids were generated by cloning the cDNA fragments for AR-V7 and AR^v567es^, respectively, into the pCMV-TurboFP635 vector. COS-7 or PC-3 cells were transfected with indicated plasmids and cultured in phenol red-free RPMI-1640 supplemented with 10% charcoal-stripped fetal bovine serum. At 40 hr after transfection, cells were pre-treated with or without 10 μM enzalutamide for 2 hr, followed by treatment with or without 1 nM R1881 for 3 hr. The COS-7 cells were then fixed with 2% paraformaldehyde, and the nuclei stained with 2.5 μM DRAQ5 (Cell Signaling). The PC-3 cells were then fixed with 70% ethanol, and the nuclei stained with DAPI. Confocal images were obtained by using a Leica TCS SP2 system with a 63X oil-immersion objective on a Z-stage, and an average of 6 fields with ~10 cells per field was captured for each group. Data quantitation was performed as described [18].

### qRT-PCR

qRT-PCR was performed as described [36]. The qPCR primer-probe sets for PSA, transmembrane protease, serine 2 (TMPRSS2), cyclin A2 (CCNA2), and UBE2C were from IDT. The primer sequences for AR isoforms were as described [13].

### ChIP and Re-ChIP

ChIP and Re-ChIP were performed as described [37]. The following antibodies were used: mouse IgG2a (ab18413, abcam), rabbit IgG (ab46540, abcam), AR-FL-specific antibody (C-terminus-directed; C-19, sc-815 x, Santa Cruz Biotech), AR-V7-specific antibody (AG10008, Precision Antibody). The PSA promoter P2-ARE primers described by Guo *et al*. [13] and the UBE2C promoter primers described by Wang *et al*. [38] were used for qPCR analysis of ChIP or re-ChIP DNA. The RPL30 exon 3 control region (Cell Signaling) was used as a negative control.

### Tumor Xenografts

Xenograft studies were conducted essentially as described [22,32]. LNCaP cells (4x10^6^) infected with lentivirus encoding AR-FL or 22Rv1 cells infected with lentivirus encoding control shRNA or AR-V7 shRNA were inoculated into castrated or intact nude mice (Charles River), respectively. The cells were mixed with 50% Matrigel and inoculated subcutaneously on the right dorsal flank. Tumor volume was calculated as *0.524 × width^2^ × length* [39]. When the tumor size reached ~100 mm^3^, the mice were randomized to daily treatment with vehicle or 10 mg/kg/day enzalutamide through oral gavage as described [22].

For the development of enzalutamide-resistant tumors, two LNCaP tumors that relapsed after enzalutamide treatment were resected, and ~20 mm^3^ pieces of the tumors were transplanted into castrated nude mice. When the tumor bits grew to 100~200 mm^3^, the mice started to receive 10 mg/kg/day enzalutamide through oral gavage. The tumors were harvested when they reached ~800 mm^3^ and serially passaged in castrated nude mice following the same protocol. The second to fourth passages of tumors were considered as enzalutamide-resistant. All animal procedures were approved by the Tulane University Institutional Animal Care and Use Committee.

### Statistical Analysis

The *Student's* two-tailed t test was used to determine the mean differences between two groups. *P* < 0.05 is considered significant. Data are presented as mean ± SEM.

## SUPPLEMENTARY FIGURES


