# Supplementary material for: Androgen receptor splice variants activating the full-length receptor in mediating resistance to androgen-directed therapy
Source: Oncotarget. 2014 Mar 4;5(6):1646–56. doi: 10.18632/oncotarget.1802 (PMC4039237; doi:10.18632/oncotarget.1802)
Supplement: Supplementary file 1 [file oncotarget-05-1646-s001.pdf]

Androgen Receptor Splice Variants Activating the Full-Length Receptor in Mediating Resistance to Androgen-Directed Therapy – Cao et al

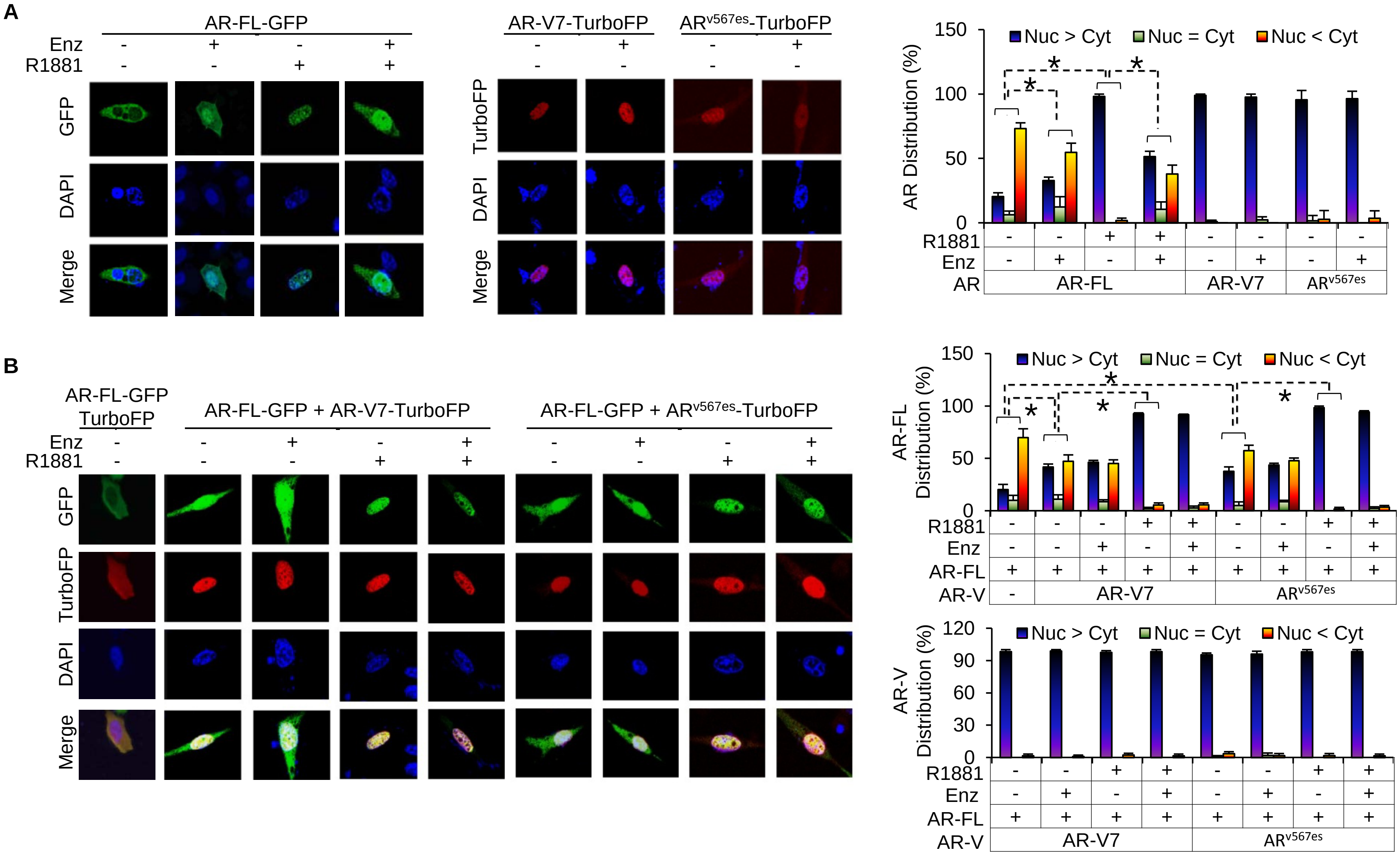

Supplementary Figure 1. Confocal fluorescence microscopy of AR-FL and AR-V localization when expressed alone (A) or when co-expressed (B) in PC-3 cells. Right panels, quantitation of % of cells with predominantly nuclear, equally nuclear and cytoplasmic, or predominantly cytoplasmic expression. DAPI, nuclear stain. Cells cultured in androgen-deprived condition were pre-treated with 10 µM enzalutamide (Enz) for 2 hr, followed by treatment with or without 1 nM R1881 for 3 hr. *, $P < 0.05$.

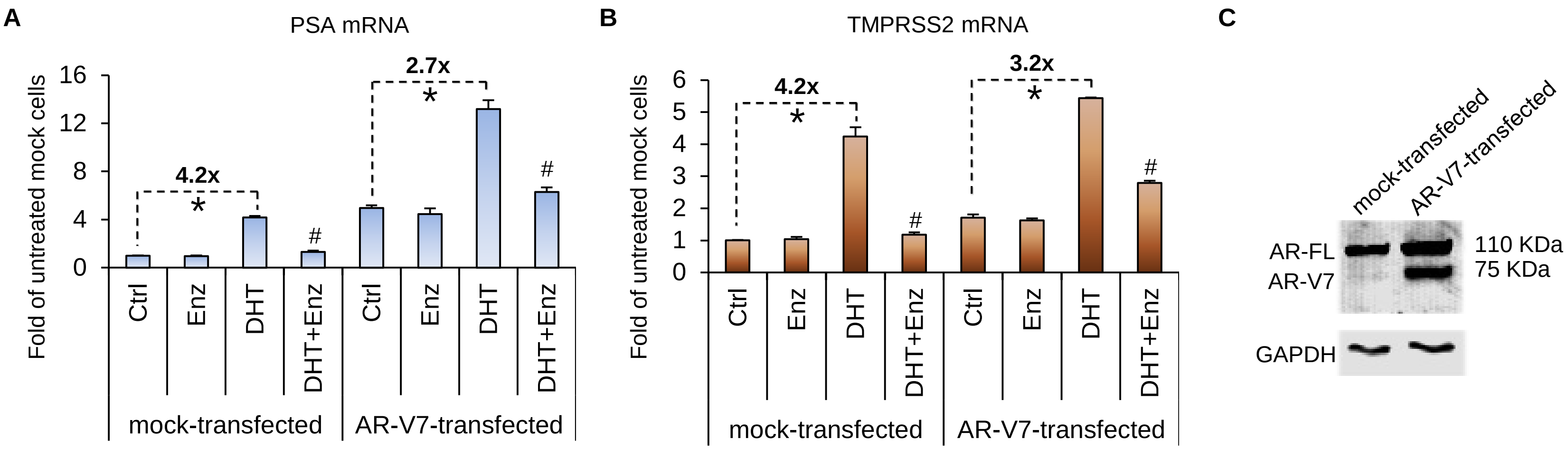

Supplementary Figure 2. AR-V diminishes the degree of response of AR-FL to androgen and enzalutamide. Inducible AR-V7-expressing LNCaP cells or mock-transfected LNCaP cells cultured under androgen-deprived condition were incubated with 30 µg/ml Cumate for 48 hr to induce AR-V7 expression and then treated with 1 nM DHT with or without 10 µM enzalutamide for 4 hr. PSA (A) and TMPRSS2 mRNA levels (B) were determined by qRT-PCR analysis, and induction of AR-V7 protein was detected by Western blotting (C). *, $P < 0.05$. #, $P < 0.05$ from DHT-treated cells.

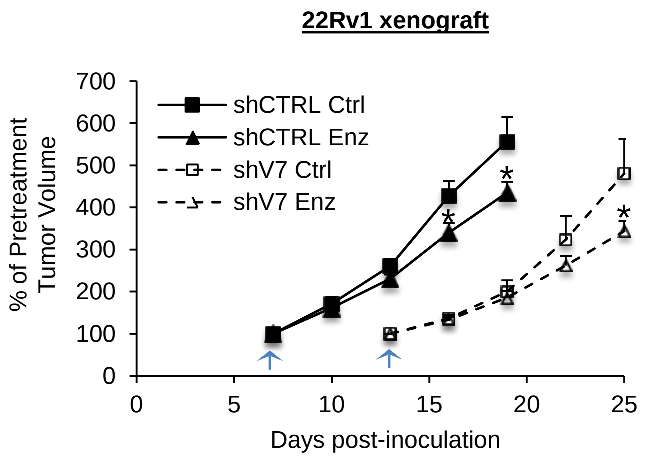

Supplementary Figure 3. Effect of enzalutamide on the growth of 22Rv1 tumors with or without AR-V7 knockdown. Arrows indicate the start of enzalutamide treatment. Data are expressed as % of pretreatment tumor volume. *, $P < 0.05$ from respective control group. Enzalutamide (Enz), 10 mg/kg/day. n = 8.

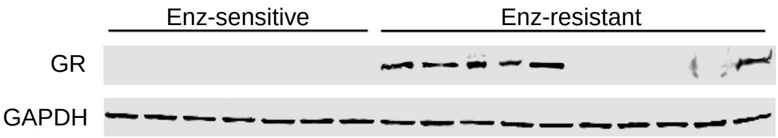

Supplementary Figure 4. Increased expression of glucocorticoid receptor (GR) protein in enzalutamide-resistant LNCaP tumors that express higher AR-V proteins.
